# In dogs does the use of bone morphogenetic proteins with internal fixation accelerates fracture healing?

**DOI:** 10.18849/ve.v10i4.722

**Published:** 2025-10-16

**Authors:** Anthi Anatolitou+, Miltiadis Markou+

**Keywords:** BONE MORPHOGENIC PROTEIN, CANINE, DOGS, FRACTURE HEALING, INTERNAL FIXATION, ORTHOPAEDIC

## Abstract

**PICO question:**

In dogs undergoing internal fracture fixation does the use of internal fixation and bone morphogenetic proteins (BMPs) accelerate healing compared to internal fixation alone?

**Clinical bottom line:**

**Category of research:**

Treatment.

**Number and type of study designs reviewed:**

Zero.

**Strength of evidence:**

Zero.

**Outcomes reported:**

There is no evidence showing that dogs undergoing fracture fixation with any internal fixation method (e.g. locking plates, dynamic compression plates) and BMPs present accelerated healing compared to internal fixation alone. In view of the absence of this evidence, it is recommended that veterinarians should base their treatment choice on their experience in internal fixation methods and BMPs usage, their available materials for the methods, the cost, the potential adverse effects, and the case-specific factors. Therefore, veterinarians should acknowledge that both methods have potential risks and complications.

**Conclusion:**

In dogs undergoing fracture fixation, there is no statistical evidence to support fracture fixation with internal fixation and BMPs as a method that accelerates healing compared to internal fixation alone.

## How to apply this evidence in practice

The application of evidence into practice should take into account multiple factors, not limited to: individual clinical expertise, patient’s circumstances and owners’ values, country, location or clinic where you work, the individual case in front of you, the availability of therapies and resources.

Knowledge Summaries are a resource to help reinforce or inform decision-making. They do not override the responsibility or judgement of the practitioner to do what is best for the animal in their care.

## Clinical scenario

You are presented with a 4-year-old Poodle with complete fractures of the right radius and ulna. You offer the treatment options available to the client. The client, an orthopaedic surgeon, prefers the option that offers the quickest rate of healing and notes that in human medicine the combination of bone morphogenic proteins (BMPs) and surgery shows promising results. You look for evidence comparing the rate of healing between internal fixation alone or combined with BMPs in order to advise appropriately.

## The evidence

There are studies that have documented faster-than-expected bone union in cases where BMPs were used, but they were excluded from the search outcome due to being case reports or articles irrelevant to the PICO question. There is no published literature directly comparing the outcomes of the two methods. Therefore, there is no evidence that dogs with fractures treated with internal fixation and BMPs show accelerated fracture healing compared with dogs treated with internal fixation alone.

## Appraisal, application and reflection

Appendicular bone fractures are one of the most common orthopaedic problems in dogs. The primary goal for any fracture treatment is to restore the normal function of the injured limb as quickly and safely as possible, including the reduction, stabilisation, and healing of the affected bones and the prevention of potential complications (Dvořák et al., 2000). There are a wide variety of fracture repair methods, depending on Association for the Study and Application of the Method of Internal Fixation (AO/ASIF) principles, the veterinary surgeon’s preference, and experience. However, several factors, such as the type, location, and severity of the fracture, along with the dog's size, age, overall health, and activity level, should be considered to determine the most suitable repair method for the specific situation. Although the repair of canine fractures has been associated with an excellent outcome due to the bone’s capacity to regain its preoperative properties (Marsell & Einhorn, 2011), the healing process can vary, and some fractures may require more extensive interventions to achieve optimal outcomes. According to one retrospective study, comminuted fractures, increased patient age, surgical site infection, and implant failure were associated with an increased likelihood of delayed union or nonunion (Marshall et al., 2022). Additionally, the study identified open fractures as being at higher risk for nonunion. Therefore, such cases may benefit from repair with a more rigid construct and regenerative methods to decrease the risk of healing complications and failures. However, Marshall et al. (2022) also reported relatively favourable nonunion rates compared to previously reported values, while emphasising the challenges in defining nonunions, which complicates the ability to predict which cases might develop nonunion and thus benefit from regenerative treatments.

Bone morphogenetic proteins (BMPs), also known as bone morphogenetic factors, are low molecular weight extracellular glycoproteins, that belong to the transforming growth factor-beta (TGF-β) superfamily and play crucial roles in various biological processes, including embryonic development, cell differentiation, and tissue regeneration (Riley et al., 1996). Bone morphogenetic proteins (BMPs) were discovered and named by Marshall Urist, who showed their innate capacity to induce ectopic bone in muscle tissue (Urist, 1965). Since then due to their osteogenic potential, BMPs have been used as therapeutical agents for managing bone fractures, periodontal defects, and regenerative medicine (Sykaras & Opperman, 2003). Bone morphogenetic proteins (BMPs) are multifunctional signalling cytokines that regulate tissue homeostasis, hence investigations of their role in diseases including osteoporosis, cancer, and cardiovascular diseases (Sanchez-Duffhues et al., 2020). They have an autocrine and paracrine mechanism of action, by binding to cell surface receptors, activating the SMAD (Suppressor of Mothers against Decapentaplegic) pathway, and regulating gene expression. Bone morphogenetic proteins (BMPs) also activate the non-SMAD pathways, contributing to their diverse biological effects (Sykaras & Opperman, 2003). Bone morphogenetic proteins (BMPs) could contribute to bone healing by promoting the recruitment and differentiation of osteoprogenitor mesenchymal stem cells into osteoblasts, enhancing osteoblast activity, stimulating angiogenesis, and facilitating the remodeling of new bone tissue (Wu et al., 2024). In human medicine, commercially available BMPs products, like recombinant human bone morphogenetic proteins (rhBMPs) promote bone proliferation in spinal fusions and non-union fractures of long bones (Ristiniemi et al., 2007). In a retrospective series study of 13 cases, Pinel & Pluhar (2012) showed that rhBMPs extra-label usage in managing delayed or non-union fractures in dogs and cats was successful without serious complications. Although there were several limitations to the study. Additionally, veterinary literature indicates that recombinant human bone morphogenetic protein 2 (rhBMP-2), a genetically engineered version of the naturally occurring BMP-2 protein, when used in combination with biomaterial matrices such as fibrin or compression-resistant matrices, may offer significant benefits in the treatment of non-union fractures. Studies such as Schmoekel et al. (2005), Verstraete et al. (2015), and Castilla et al. (2023) demonstrate that rhBMP-2 enhances bone healing in dogs and cats with chronic and non-union fractures, which are often resistant to conventional treatment methods. The ability of rhBMP-2 to stimulate osteogenesis and promote bone regeneration makes it particularly effective in these challenging cases. Given the promising results, it is crucial to highlight the potential of rhBMP-2 in non-union fracture healing, suggesting the need for further research and a comprehensive Knowledge Summary on its long-term efficacy and optimal usage in these clinical settings. However, the cost of rhBMPproducts, is a major limitation of their usage.

Bone morphogenetic proteins (BMPs) can be applied directly to the canine fracture site or surgical area, often in a carrier matrix that maintains the BMPs in place and provides a scaffold for new bone growth (Tuominen et al., 2001). Additionally, BMPs can be incorporated into biodegradable scaffolds implanted at the site of injury. These scaffolds gradually degrade, releasing BMPs and providing structural support for new bone formation (Toriumi et al., 1991). Also, BMPs can be administered as part of an injectable formulation, delivering the proteins directly to the injury site (Zhu et al., 2022). In veterinary medicine the usage of BMPs remains limited, especially due to immunogenicity, difficulty in achieving proper structure and stability in solutions, and cost (Zygmuntowicz et al., 2020).

According to literature research, no evidence directly addressed the PICO question. Although published studies have investigated BMPs’ efficacy in regenerative therapies for bone defects, no studies published in English have specifically compared the use of internal fixation with and without BMPs regarding fracture healing time. Two studies published in Portuguese compared the outcomes of treatment for canine radio-ulnar fractures using bone plates and screws, with and without the addition of BMPs (Ferrigno et al., 2007; Nina et al., 2007). However, both studies have only their abstract published in English, therefore they did not meet the inclusion criteria for this PICO.

Comparatively, in humans, the Food and Drug Administration (FDA) has approved only two genetically engineered rhBMP formulations—rhBMP-2 and rhBMP-7—for the treatment of certain orthopaedic conditions, including open fractures, nonunion fractures, vertebral fusion, and maxillofacial bone augmentation (Medtronic Sofamor Danek, 2002.) In veterinary medicine, other options for accelerating bone healing, such as autologous bone grafting (Harasen, 2011), platelet-rich plasma (López et al., 2019), stem cell therapy (Anatolitou et al., 2021) or omental grafting (Ree et al., 2018), may serve as viable alternatives to BMPs, especially in cases where a more established approach is preferred. These techniques promote healing by enhancing vascularisation, improving blood supply, and stimulating the regenerative process at the fracture site.

In view of the absence of evidence, it is recommended that veterinarians should base their treatment choice on their experience in internal fixation methods and BMPs usage, their available materials for the methods, the cost, the potential adverse effects, and the case-specific factors. While generally safe, BMPs can sometimes cause excessive bone growth or inflammatory reactions at the site of application (May et al., 2019). Also, considering that a large number of primary osteosynthesis cases result in union without complications, with non-union rates ranging from 3.4% to 8.1% in veterinary literature (Marshall et al., 2022), the cost-benefit of using BMPs in primary osteosynthesis cases should be carefully considered.

Considering the benefit the answer to this PICO question represents for veterinary clinicians in practice, further studies should be designed ideally as double-blinded, controlled, randomised clinical trials comparing specifically the use of internal fixation with and without BMPs required to collect data regarding fracture healing time. Until now, numerous publications suggest that healing occurs within or faster than the typical timeframes expected in the absence of BMPs. Specifically, these studies have been conducted in various species: dogs (Murakami et al., 2003; Pinel & Pluhar, 2012; Massie et al., 2017; Park et al., 2018; Lee & Kang, 2024), dogs and cats (Schmoekel et al., 2004; Pinel & Pluhar, 2012), and across a range of experimental models including mice, rats, rabbits, dogs, sheep, and non-human primates (Stokovic et al., 2021). While these studies indicate potentially accelerated healing in certain cases, they do not provide direct comparisons between BMPs-treated and non-BMPs-treated fractures under controlled conditions. Also, a lot of them are case reports, therefore provide the lowest level of evidence in the evidence-based medicine pyramid (Pinel & Pluhar, 2012; Park et al., 2018; Lee & Kang, 2024). Moreover, future studies should be accompanied by robust statistical analysis. Also, larger sample sizes based on power analysis and longer-term outcomes are needed to determine if there is any difference between internal fixation of bone fractures with BMPs and without BMPs regarding fracture healing time. Furthermore, randomised clinical trials should be performed to address biological processes and minimise the effects of confounding variables. These studies will provide insight into the real potential of this biological approach to favour bone healing. In conclusion, there is currently no evidence that canine bone fracture healing is more rapid when treated with internal fixation with BMPs versus without BMPs.

## Methodology

**Search Strategy:** 

Databases searched and dates covered:	CAB Abstracts on OVID interface 1973 to 2025 Week 01 PubMed accessed via the NCBI website 1920 to January 2025
Search strategy:	CAB Abstracts: (dog or dogs or canine* or canis).mp. ((internal fixat* or plat* or screw* or pin* or wire*) and (bone morphogenic protein or BMP)).mp. (fractur* or bone*).mp. 1 and 2 and 3 PubMed: dog OR dogs OR canine OR canis (internal fixation OR plate OR screw OR pin OR wire) AND (bone morphogenic protein OR BMP) fracture OR bone 1 AND 2 AND 3
Dates searches performed:	08 January 2025

**Exclusion / Inclusion Criteria:** 

Exclusion:	Irrelevant to the PICO. Articles not in English.
Inclusion:	Experimental studies.

**Search Outcome:** 

Database	Number of results	Excluded – article relevant to the PICO	Excluded – article not in English	Total relevant papers
CAB Abstracts	11	9	2	0
PubMed	26	26	0	0
Total relevant papers when duplicates removed				0

## ORCiD

Anthi Anatolitou: https://orcid.org/0000-0002-8705-3249

Miltiadis Markou: https://orcid.org/0000-0001-9754-2057

## Conflict of Interest

The author declares no conflicts of interest.
